# 12 Tips for Creating High Impact Clinical Encounter Videos - with Technical Pointers

**DOI:** 10.15694/mep.2019.000092.1

**Published:** 2019-04-18

**Authors:** Ge Shi, Siyoung Lee, Yue Yuen, John Liu, Zachary Rothman, Paul Milaire, Stephen Gillis, Alexandre Henri-Bhargava

**Affiliations:** 1 University of British Columbia; 2 MedIT - University of British Columbia; 3 University of British Columbia - Island Medical Program

**Keywords:** Cognitive Theory of Multimedia Learning, Video, Medical Education, Clinical Skills, Neurology, Video Editing, Learning Theory, Flipped Classroom, Pedagogy, Medical Teaching

## Abstract

This article was migrated. The article was marked as recommended.

Videos are increasingly used in medical education. They are effective for teaching difficult-to-grasp concepts that rely heavily on visuospatial processing ability such as anatomy, surgical procedures, and physical exam maneuvers. Common pitfalls of existing videos include lackluster audiovisual quality, poor camera angles, absence of formal teaching as narration, or excessive length. This article serves to assist educators who wish to produce educational clinical encounter videos that maximize student learning. We detail 12 tips focused on improving the quality of clinical educational videos, mitigating cognitive load within a video, and understanding the technicalities of video production. These tips are based on review of existing literature on neurocognitive learning theories and the succeeding Cognitive Theory of Multimedia Learning (CTML), as well as our experience in producing educational videos.

## Introduction

With advancements in technology, there is a growing number of videos designed to heavily supplement or completely replace traditional textbook- and lecture-based learning in educational curricula, such as in massive open online courses ( Breslow *et al.*, 2013) and “flipped-classrooms.” This paradigm shift also exists in medical education and is not only attributable to the increased use of digital media in society at large, but to the ever-expanding knowledge base of medicine that requires more efficient teaching and learning strategies ( Prober and Khan, 2013).

Currently, there are few of pedagogical videos focused on clinical encounters. Popular medical videos such as Khan Academy ( www.khanacademy.org) focus on elucidating the pathophysiology of diseases, diverting students’ attention away from the importance of clinical encounters ( Prober and Khan, 2013). Clinical videos simulate real patient encounters with a focus on recreating the clinical environment, visually capturing physical exam maneuvers, and displaying normal and abnormal physical findings. This integrates relevant physiology teaching through narration and animation and helps create mental imagery, ultimately reinforcing relevant medical knowledge into memory ( de Leng *et al.*, 2007).

Video design should be based on evidence-based neurocognitive learning theories such as the Cognitive Theory of Multimedia Learning ( Mayer and Moreno, 2003). The 12 tips we provide focus on technical considerations when creating a clinical video, implementation of multimedia learning theories to bolster student learning, and the nuances of teaching a clinical encounter through motion picture.

## Tip 1: Perform a needs assessment

An important initial consideration is whether a video is the appropriate medium for delivering your content. Dense, didactic content may be better left in text format due to the resource-heavy nature of creating a video ( Norman, 2017). Visuospatial-heavy skills such as laparoscopic surgery ( van Det *et al.*, 2011) and physical exam techniques, however, are better demonstrated through video.

Subsequently, assess the strengths and weaknesses of existing videos, paying attention to topic, audiovisual quality, volume and depth of content, length, and availability ( Dong and Goh, 2014). In the age of information overload, gauge if your video is an improvement on existing medical education videos.

The audiovisual quality of educational videos is crucial but often overlooked. If poor, it can impede learning ( Sweller, 1988; Mayer and Moreno, 2003; Kalyuga, Chandler and Sweller, 2004). However, creating high production value videos are a time- and money-draining endeavour for an academic clinician ( Brownell and Tanner, 2012; Hansch *et al.*, 2015). A deterrent to developing online materials including videos is lack of faculty time (Shea, 2007; Lloyd et al., 2012; Allen and Seaman, 2016). A common strategy is to recruit students who will reduce the burden of time and cost. Furthermore, they offer a valuable perspective as the target audience, and they themselves benefit by learning the content by teaching ( Moore, 1973).

## Tip 2: Factor in cognitive load

Drawing upon a number of existing theories of learning, the cognitive theory of multimedia learning (CTML) models how the human brain processes multimedia ( Mayer and Moreno, 2003). CTML describes how visual and auditory information are processed in separate streams and states that they are limited by working memory ( Sweller, 1988). The total burden on working memory when watching a video is called the *cognitive load* of the video. Cognitive load is the sum of: (1) *intrinsic load*, the inherent difficulty of the material; (2) *germane load*, the processing power necessary to make sense of new information and integrate it into previous knowledge; and (3) *extraneous load*, the burden from unnecessary distractions in the video. Therefore, student learning can be maximized by managing these three appropriately ( Table 1).

**Table 1.** Cognitive load modification strategies to increase student learning

Method of Modification	Definition
Chunking	Grouping individual items into one larger unit.
Signalling	Directing attention to key ideas by visual or auditory means.
Synchronizing	Integrating both visual and auditory modalities in teaching a concept.
Aligning	Placing graphics & related text spatially close to one another to reduce the need for viewer’s eyes to scan the screen.
Eliminating Redundacy	Avoiding repetition of material in both the visual and auditory streams.
Weeding	Eliminating extra information that is not the pertinent focus of the material.
Off-loading	Divising material between the visual and auditory streams so that one stream is not overloaded.

Further details on modifying cognitive load are discussed in tips 8 and 9.

## Tip 3: Choose audience-appropriate content for optimal engagement

The effectiveness of an educational video depends on its ability to retain learner attention. In one of the largest video engagement studies to date with data collected from 6.9 million sessions, video length was the biggest predictor of viewer engagement ( Guo, Kim and Rubin, 2014). Many studies show that viewer attention begins to taper off after around 6 minutes. Knowing your audience will allow you to tailor your content and implement *weeding* ( Table 1) to keep your video short. It may be tempting to include every detail, but remain judicious and remember that you are doing a disservice to learners by unnecessarily prolonging the length of the video, losing their attention, and overloading their working memory with *extraneous load* **.**

## Tip 4: Organize your pre-production documents: script, storyboard, shot list, shot map

The planning phase has the largest impact on the video’s engagement level ( Guo, Kim and Rubin, 2014). Organizing your content into pre-production documents will increase production and post-production efficiency.

*Script* A script is a preliminary content document that serves as a foundation for the storyboard. It should be written according to your learning objectives to ensure all pertinent content is covered.

*Storyboard* A storyboard (Supplementary 1) is a master document which divides the content from the script, and temporally organizes it into side-by-side columns: action (visual component), speech (audio component), and cinematography/post-production. This helps those involved in production to visualize what the video will look like at every second.

This organization facilitates *off-loading* and *synchronizing* ( Table 1). By ensuring that every teaching point has a visual and corresponding auditory component, you can prevent overloading of one stream. Effective *off-loading* and *synchronizing*can be achieved by adding voice-overs while clinical manoeuvres are being performed on-screen or inserting animations to illustrate a point.

The cinematography/post-production column details camera shots required to capture the action in the most visually pleasant and effective way possible. (More on effective shots in Tip 9). It also include notes on post-production editing, which will be helpful in the editing phase.

*Shot list* Storyboards are organized in a chronological order. However, to facilitate a practical filming day and decrease the time required to move equipment, re-sort the scenes into a shot list (Supplementary 2) which is based on room setup: camera lens and position, location, availability of actors, and equipment location. Then film based on the priority of the shots.

*Shot map* A shot map is a bird’s-eye view of the room that shows the equipment and different camera angles (Supplementary 3) which is a useful reference for room set-up and filming. Assign letters to each camera angle and reference them in the shot list and storyboard.

## Tip 5: Choose appropriate actors

*Doctors* It is helpful to find clinicians with good articulation who are experts in the topic of the video. Viewers pick up tips and tricks through observations, such as ways of positioning patients that best elicit the tested response, style of holding equipment, and organization of reporting findings.

*Actors/Patients* Choose a patient or actor who best demonstrates the desired physical exam traits. For example, if trying to demonstrate a pupillary light reflex, it is best if the patient has light-coloured irises so there is better contrast between the pupil and iris. To prevent finding incidental “abnormal” physical exam findings on the day of filming, screen the patient/actor beforehand.

If the objective of the video is to present pathology, try to find a patient with the condition, as mimicking pathologies may look unnatural and depreciate the overall impression of the video. If a patient with the condition cannot be found, alternatives include using a pre-existing video demonstrating the pathology, or acting out the finding, stating it is an actor’s representation of the condition to the best of their ability.

## Tip 6: Streamline the filming day

Evaluate your budget and access to video filming resources such as equipment and members with video-production skills. Many institutions have an IT or Media department that can provide equipment and technical support, as well as general guidance for creating digital educational content. Alternatives include purchasing ( Table 2) or renting equipment.

**Table 2.** Standard cinematography equipment with associated costs

Cost	Low ($)	Medium ($$)	High ($$$)
Video Camera	Cell phonePoint & shoot digital cameras	DSLR	Cinema Camera
Stabilization	HandheldFurniture & makeshift	TripodWeighted manual stabilizer	Motion stabilizersDolliesSliders
Lights	Natural light: window, outside	Household or construction site lights	Video production lights
Audio Recorder	Cell phoneBuilt-in mic in camera	Microphone	Wireless lav mic

All prices are in CAD. Low ($): <$400. Medium ($$): $50 - $2,000. High ($$$): +$1,000.

*Filming site* Pick a sound-proof location such as clinical examination rooms in hospitals or teaching units in University buildings ( Dong and Goh 2014). This minimizes distracting background noise and is reflective of the private and safe clinical environment. Tape signs to let passers-by know there is filming in progress. Never underestimate the amount of space required, as it will be crowded with equipment and people on filming day. Try to remove reflective surfaces or distracting objects on walls and floors.Filming sets can be hazardous with wires, bars, and different kinds of stands and equipment. Organization and cleanliness on the day of filming will greatly facilitate efficiency.

*Division of Roles* With the luxury of students or helping hands, role division can greatly increase the quality and efficiency of filming day.

• *Director.* Important overviewing role in charge of tone, flow, and organization of the day. Decides whether to re-take a shot or to move on. Observes for any distracting habits that actors may have (e.g. clearing throat, looking to the director during scenes). Must consider the crew’s comfort level, fatigue, hunger, and attitude throughout filming.
• *Camera person.* Responsible for operating camera and mic and assure audiovisual quality. Responsible for creating a back-up of the video after filming. Realizing there is missing or improper footage after-the-fact can be devastating, as finding the time and resources to re-film can be a hurdle in production.
• *Continuity person.* Records details about props, lighting, and actors’ movements to ensure consistency between different takes of one scene as well as in between different scenes. Maintaining continuity reduces the visual distraction for viewers and is a form of *weeding.*

*Filming* The general rule is to film more coverage than needed (multiple angles, additional takes). If possible, use two cameras simultaneously, with different camera angles or positions. This saves time and ensures continuity. Ensure all your shots are in focus.

## Tip 7: Follow common filming practices

*Frame your shot* Generally, aim for the smallest frame which will capture what is necessary for your scene. For example, if the clinical exam involves the upper body, it is not necessary for the legs to be in frame. Follow the rule of thirds to create an aesthetically pleasing frame, and leave enough room above the heads of the actors.

*Angles* The 180-degree rule delineates that when filming two subjects and changing views, the camera angles should not exceed 180 degrees from each other to maintain a consistent point of view for viewers. The 30-degree-rule states that when changing angles the camera angles should be at minimum 30 degrees apart from each for a change in angle to be worthwhile.

*Panning* When filming, anticipate the movements of participants/actors. For scenes where large movements are required, either be prepared to pan with the movements, or film with a wider lens or camera set-up further away for a wider frame.

*Cutting with purpose* Cutting between scenes or angles can be used as a form of *signalling.* Cuts which are made at the maximal point of intensity of a scene, such as when the demonstrator reaches over to test the patient’s muscle, can highlight and draw attention to the importance of that particular movement.

## Tip 8: Edit your video while adhering to principles of CTML

**Figure 1.**
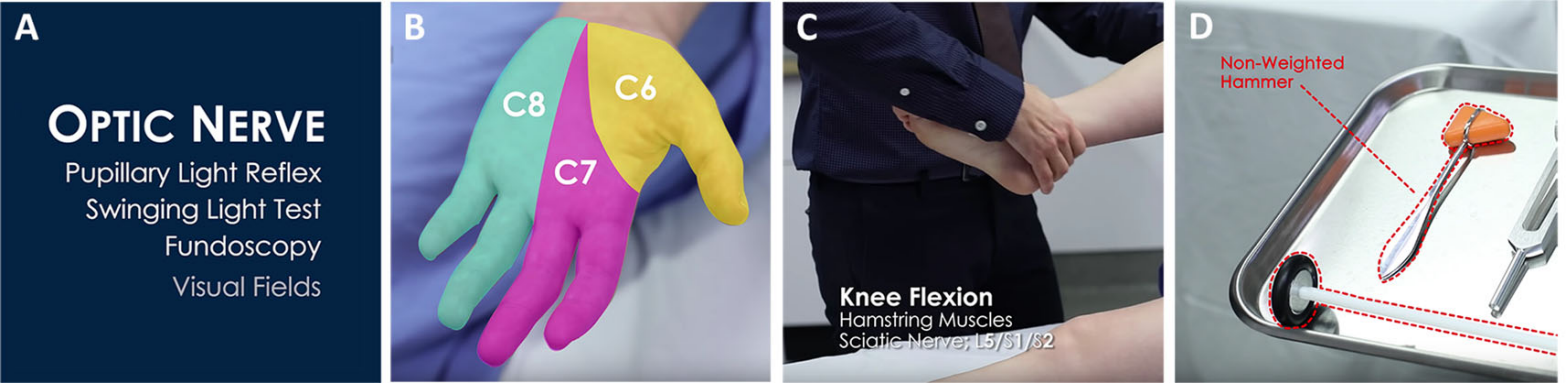
Examples of Video Editing Techniques. A. *Chunking* by dividing a video with title screens. B. *Aligning* where color and text overlay work together to show distribution of 3 distinct spinal nerves. C. Stroke function used to improve readability of font. D. *Signalling* class of reflex hammer.

To organize your video clips, name them based on the pre-created shot list labels to maintain consistency and minimize confusion. When editing (see Supplementary 4 for softwares), avoid distracting texts or animations. Be consistent with colour schemes, font, and formatting of words. To avoid difficult to read text overlaying a background, use a stroke or shadow function ( Figure 1.C). Ensure there are adequate (2.5 cm) border margins between figures and the edges of the video.

*Chunking* Chunk topics into separate videos to provide a natural break between concepts. Divide your video content into additional chunks separated by title screens ( Figure 1.A).

*Signalling Signalling* can be achieved by editing shot transitions or adding animation. It can be as simple as pointing to what is being discussed. For example, outlining the reflex hammer when explaining it ( Figure 1.D).

*Aligning* Any on-screen text or graphics should supplement the viewer’s understanding by reducing cognitive load through *aligning*, and not overburden the viewer with redundant or distracting information ( Figure 1.B).

## Tip 9: Record and edit audio effectively

*Audio Recording* Ideal audio recording is crisp and clean. Noise protection filters (pop filters, wind screens) dampen distracting high frequency noises, which reduces cognitive load through *weeding.* Use narrators and actors who have a voice that is pleasing and soothing for all listeners. A conversational and enthusiastic tone, rather than an over-rehearsed and robotic tone, can enhance learners’ engagement with the video ( Guo, Kim and Rubin, 2014; Guy, Byrne and Rich, 2014; Rana, Besche and Cockrill, 2017). Students also engage better with faster-speaking instructors ( Guo, Kim and Rubin, 2014).

*Audio Editing* See Supplementary File 4 for audio software. *Synchronizing* ( Table 1) of audio and visual components can be achieved by simultaneously editing the audio as you view the video. Normalize audio levels between scenes for consistency and fade in and out audio segments to reduce distractions.

*Music* If you choose to include music, do so with purpose, as it can be distracting if used unnecessarily. If used, music should be euphonic and harmonious, and can effectively hide mic scratching or background ventilation noise, as well as provide a soothing atmosphere. Cacophonic music can be jarring and take attention away from video learning. Remember that the video may be viewed by a variety of demographics internationally when uploaded online.

## Tip 10: Choosing the right sharing platform

Careful consideration for how and where to share your video is important as there exist a plethora of hosting platforms, each with their pros and cons. If you reference a local resource in the video (i.e. university-specific lecture hand-out), it may be appropriate to host your video privately for students of that locale. However, if significant resources are invested for high production and lasting educational value for as many students as possible, then consider public international host websites.

Studies show that perceived benefits in learning are correlated with customizability: ease of use ( Kay, 2012), and ability to rewind, rewatch, and fast forward in a “flipped-classroom” setting ( Cook et al., 2008; Baker, 2011; Prober and Heath, 2012).

Additional considerations include:

• Cost of hosting
• Hyperlinking possible to direct students to certain timestamps for ease of reference
• Built-in download functionality
• Password protection for sensitive content
• Ability to control comments & likes/dislikes (helpful to prevent inappropriate anonymous commentary)
• Monetization of your video via ads
• Metadata and statistical tracking

## Tip 11: Beware of legal guidelines

*Consent forms* Institutions often have pre-written media consent forms that you can rework. Consider your intended video hosting platform, as clinicians/actors/patients may consent to institution-wide sharing but not international distribution.

*Hosting Servers* Clear any conflicts of interests or information-hosting guidelines as per your institution. Certain institutions do not allow the hosting of information in an out-of-country server and some have pre-existing agreements with certain companies. It is important to abide by hosting guidelines as infringement will result in removal of content.

*Audio Copyright* With an abundance of music easily downloadable from the internet, it is possible to mistakenly use music that has been copyrighted. Avoid incidental copyright infringement and risking getting your video removed by using free creative commons sources, such as freemusicarchive.org.

*Long-Term Ownership* Once published, a person or a group must take ownership and responsibility of the video for the future. On a public host server (such as YouTube), there may be issues that arise such as copyright infringement, other channels re-uploading your video without consent, or hyperlinks included in your video requiring updating in the future. Long-term faculty and IT departments are ideal for assuming long-term responsibility of these videos.

## Tip 12: Evaluate effectiveness of video

To overcome common low response rates on feedback surveys, embed the video within a survey form so students do not have to click an extra link to provide feedback. Place the video between pre- and post-viewing questions (“How knowledgeable are you about this topic?”) to assess the subjective amount of learning the video provided. Additionally, word questions in a direct and active manner such as “Describe one thing that you would improve” as opposed to “Is there any improvement you would suggest, if so what?” Free comment feedback can provide valuable information on qualitative improvements to your video.

Feedback responses are often polarized to extreme likes and dislikes. To mitigate this selection bias, metadata and video statistics can be used to analyze all views and viewers of a video. Many hosting sites provide statistics to the content uploader such as number of total persons who viewed the video, average time of a viewing, demographic of the audience (age, gender, location), and if viewing patterns change temporally. This is an often overlooked tool to assess effectiveness of the video but can supplement standard feedback survey responses.

## Conclusion

As videos are incorporated into educational curricula, a greater emphasis will be placed on quality and ability to efficiently and effectively teach students in a flipped-classroom setting. The 12 tips we provide are designed to guide medical educational video production, with focus on integrating educational theory (CTML), the technicalities of video production, and illustrating clinical encounters.

## Take Home Messages

• Perform a needs assessment prior to creating a video to gauge if your video is an improvement on the current existing medical education videos.
• Incorporation of theories of learning, such as the Cognitive Theory of Multimedia Learning, improve the efficacy of student learning from videos.
• Pre-production documents such as the script, storyboard, and shot list are cinematography tools which can help organize your content and greatly enhance the efficiency of filming day.
• Edit your video adhering to theories of learning to maximize efficacy of learning and minimize the dead time.
• Ensure there is long-term responsibility for the video as there may arise copyright or other issues that may require attention in the future.

## Notes On Contributors

**Shi, Ge** is part of the MD graduating class of 2020 at the University of British Columbia at the Vancouver Fraser Medical Program.

**Lee, Siyoung** is part of the MD graduating class of 2020 at the University of British Columbia at the Vancouver Fraser Medical Program.

**Yuen, Yue Ting** is part of the MD graduating class of 2020 at the University of British Columbia at the Vancouver Fraser Medical Program.

**Liu, John** is part of the MD graduating class of 2020 at the University of British Columbia at the Vancouver Fraser Medical Program.

**Rothman, Zachary** is a Senior Producer and Creative Lead at MedIT at University of British Columbia’s Faculty of Medicine.

**Gillis, Stephen** is an Associate Producer at MedIT at University of British Columbia’s Faculty of Medicine.

**Milaire, Paul** is a Technical Producer at MedIT at University of British Columbia’s Faculty of Medicine.

**Dr. Henri-Bhargava, Alexandre,** MD, CM, MScCH, FRCPC is a Clinical Associate Professor of Medicine (Neurology) at the University of British Columbia, based at UBC’s Island Medical Program (IMP) in Victoria, Canada. He is the site-specific discipline leader for neurology at the IMP. He is the Medical Director of the Neil and Susan Manning Cognitive Health Initiative.

## Declarations

Dr. Henri-Bhargava has received clinical trial support from the Canadian Consortium on Neuro-degeneration in Aging, Hoffman-Laroche, Intelgenx, TauRx, Eli Lilly, Boehringer Ingelheim, AstraZeneca, and Genentech. None of these trials are pertinent to medical education or the subject matter of this paper. The authors alone are responsible for the content and writing of the article, the knowledge gleaned largely from the creation and distribution of their own multimedia resources for Neurology Clinical Skills teaching at UBC.
