# Supplementary material for: 12 Tips for Creating High Impact Clinical Encounter Videos - with Technical Pointers
Source: MedEdPublish (2016). 2019 Apr 18;8:92. [Version 1] doi: 10.15694/mep.2019.000092.1 (PMC10712636; doi:10.15694/mep.2019.000092.1)
Supplement: Peer review history

# Reviewer report: Reviewer response for version 1

**DOI:** 10.21956/mep.19803.r29796

**Published:** 2019-07-14

**Authors:** Ken Masters

**Affiliations:** 1 Sultan Qaboos University

This review has been migrated. The reviewer awarded 4 stars out of 5 This is a useful introduction and guide to creating clinical encounter medical education videos. The paper focuses on the basics. Some more advanced users may find this too elementary, but many of these basics are the things that are frequently overlooked, by newbies as well as those who have been creating videos for some time. The authors have also found a good balance between theoretical concepts and practical experience for their source of information, and the support of the supplementary files is also useful.Although some greater emphasis could be placed on the managerial and evaluation aspects of the processing, one must also see the creation of such videos within the context of education institutions' current practice of rarely demanding that lectures notes and presentation slides undergo formal evaluation and assessment. The authors should add a note, however, indicating that some institutions may have educational protocols regarding teaching materials, especially their relationship to the curriculum, language suitability for their students, etc., and so video creators should have the required permissions and approval before and during the creation period. This is primarily because post-production editing may not always be feasible on some of these issues.It would also have been useful if the authors would encourage the creation of sub-titles, whether embedded or as a separate file, as this will add great value to any instructional video. If the video creators are intending to have sub-titles, however, they should take this aspect into account from the outset when framing their subjects.Overall, however, a useful guide.

Reviewer Expertise:

NA

No decision status is available

# Reviewer report: Reviewer response for version 1

**DOI:** 10.21956/mep.19803.r29794

**Published:** 2019-04-19

**Authors:** P Ravi Shankar

**Affiliations:** 1 American International Medical University

This review has been migrated. The reviewer awarded 4 stars out of 5 Thank you for the invitation to review this interesting paper. Videos are becoming an increasingly important part of education and are increasingly preferred by the current generation of students. A number of learning platforms provide access to good quality videos. My experience with creating videos has been limited to platforms which synchronize video and audio recording using a webcam or the computer camera and microphone with screen capture functionality. The twelve tips for creating videos have been well described. The authors have tried to relate their description with the theories of cognition. They have addressed a number of areas many of which may be unfamiliar to many faculty members and may require more detailed description. I am happy to note that many of the authors and medical students. The supplementary files are interesting. The tables aree also useful. I am of the opinion that commercial software which combines inputs from a camera, microphone and screen capture will allow an affordable option to create good quality videos especially for institutions which lack access to a good audiovisual department. All medical educators will find this article to be of interest.

Reviewer Expertise:

NA

No decision status is available

# Reviewer report: Reviewer response for version 1

**DOI:** 10.21956/mep.19803.r29795

**Published:** 2019-04-18

**Authors:** Lee HangFu

**Affiliations:** 1 Windsor University School of Medicine

This review has been migrated. The reviewer awarded 3 stars out of 5 I had the opportunity to review this paper titled, "12 Tips for Creating High Impact Clinical Encounter Videos - with Technical Pointers". The authors have provided important tips on video production and post-production process. Many of the pitfalls have been identified, however, I would like to point out a few more important issues that should be discussed in the paper: 1) The support from the Institution; 2) The standardization of the Expert physician to the practical techniques as according to the Learning Objectives (LO) of the curriculum; 3) Standardization of the actor/Simulated Patient to the presenting pathological Signs and Symptoms of the disease; 4) a formative assessment to evaluate the reliability and validity of the video content learned to the LO specifications. It is a valuable paper but the needs for standardization, reliability, and validity must be considered in the video pedagogy.

Reviewer Expertise:

NA

No decision status is available
